# CD117 expression in operable oesophageal squamous cell carcinomas predicts worse clinical outcome

**DOI:** 10.1111/his.12111

**Published:** 2013-02-11

**Authors:** Huijie Fan, Yuan Yuan, Junsheng Wang, Fuyou Zhou, Mingzhi Zhang, Karl-Erik Giercksky, Jahn M Nesland, Zhenhe Suo

**Affiliations:** 1 Department of Oncology, The First Affiliated Hospital of Zhengzhou UniversityZhengzhou, Henan Province, China; 2 Department of Pathology, The Norwegian Radium Hospital, Oslo University Hospital, University of OsloMontebello, Oslo, Norway; 3 Department of Pathology, Capital Medical UniversityBeijing, China; 4 Department of Oncology, Anyang Tumour HospitalAnyang, Henan Province, China; 5 Department of Surgery, Anyang Tumour HospitalAnyang, Henan Province, China; 6 Department of Surgery, The Norwegian Radium Hospital, Oslo University Hospital, University of OsloMontebello, Norway; 7 Department of Pathology, Institute for Clinical Medicine, Faculty of Medicine, University of OsloOslo, Norway

**Keywords:** cancer stem cell, CD117, immunohistochemistry, oesophageal squamous cell carcinoma, stem cell factor

## Abstract

**Aims:**

To investigate the aberrant expression of CD117 in oesophageal squamous cell carcinoma (SCC) and its prognostic significance.

**Methods and results:**

Immunohistochemical staining for CD117 was performed on tissue microarray and routine tissue sections from 157 oesophageal SCC patients and 10 normal oesophageal epithelia adjacent to tumour. The positive rate of CD117 expression was 29.9% in oesophageal SCC tissues, whereas no CD117 expression was detected in the 10 normal oesophageal epithelia. CD117 expression was significantly associated with T stage (*P* < 0.001), distant metastasis (*P* = 0.015), lymph node metastasis (*P* = 0.019), and clinical stage (*P* = 0.021). Progression-free survival in the patients with CD117-positive tumours was shorter than that in the patients with CD117-negative tumours (*P* = 0.010). In univariate analyses, CD117 expression was the most significant factor for overall survival of oesophageal SCC patients (*P* < 0.001), followed by lymph node metastasis (*P* = 0.001), T stage (*P* = 0.002), clinical stage (*P* = 0.006), distant metastasis (*P* = 0.020), and histological grade (*P* = 0.027). Multivariate analyses verified that CD117 expression was an independent prognostic marker for oesophageal SCC patients (*P* = 0.002). In addition, CD117 expression predicted poorer survival in patients without distant metastases.

**Conclusions:**

CD117 expression in operable oesophageal SCC may be a valuable prognostic marker, and detection of its expression in clinical samples may be useful in defining a subclass of oesophageal SCCs with extremely poor clinical outcome, which may require a specially targeted treatment modality.

## Introduction

Oesophageal cancer is the eighth most common malignancy worldwide, and ranks sixth as the most frequent cause of cancer-related deaths, accounting for nearly 4% of all male malignancies.^1^ Among all histological subtypes, squamous cell carcinoma (SCC) is the major type in Asia, constituting approximately 90% of oesophageal cancers.^2^ Survival rates after oesophageal cancer diagnosis still remain poor, despite standardization of surgery and adjuvant treatment for resectable tumours.^3^ Identifying powerful prognostic markers, so as to improve the survival of oesophageal SCC patients by better targeting of therapy, is currently a major challenge for both clinical practitioners and pathologists.

CD117, encoded by the proto-oncogene c-*kit*, is a transmembrane protein belonging to the type III subfamily of the receptor tyrosine kinases.^4^ It has extracellular, intramembranous and intracellular domains. By binding to its ligand, called stem cell factor (SCF), this molecule plays an important part in regulating cellular activities, such as apoptosis, cell differentiation, proliferation, and cell adhesion.^5^ Expression of CD117 has been described in numerous normal cells, including haematopoietic cells, germ cells, mast cells, melanocytes, breast epithelial cells, and Cajal cells of the gastrointestinal tract.^6^ In addition, it has been documented in various tumours, including mast cell leukaemia, Ewing sarcoma, neuroblastoma, melanoma, thyroid, endometrial, ovarian and breast cancers, and small-cell lung cancers.^7^–^15^ Notably, CD117 is a positive diagnostic marker of gastrointestinal stromal tumour (GIST), and is widely used in oncology treatment guidelines.^16^

Despite its wide expression in various normal and malignant tissues, the expression of CD117 and its clinical role in oesophageal SCC remain unclear. To the best of our knowledge, CD117 expression status in oesophageal SCC has not been reported in English-language journals. In this study, immunohistochemical analysis of this molecule was performed in a large number of surgical specimens (*n* = 157) from oesophageal SCC patients within a geographical area (Anyang, China) where the incidence of this cancer is notably high. Clinicopathological correlations with CD117 expression in the tumours of these patients with 10 years of follow-up were investigated.

## Materials and methods

### Patients and materials

This study was a collaboration between Anyang Tumour Hospital, Henan, China, and the Norwegian Radium Hospital of Norway, which was approved by the Ethics Committee of Anyang Tumour Hospital and Anyang Hygiene Bureau. Oesophageal SCC patients were eligible if they underwent potentially curative surgery without preoperative chemotherapy or radiotherapy during the period 1989–1994 at Anyang Tumour Hospital, Henan, China. One hundred and fifty-seven patients met the eligibility criteria. Among these patients, 95 were men and 62 were women, ranging from 33 to 73 years of age, with a median age of 57 years. All tumours were classified according to the International Union against Cancer (UICC) 2003 standard.^17^ Surgically removed specimens were routinely fixed in buffered formalin and blocks embedded in paraffin blocks for clinical diagnosis; tumours were reclassified for this study. In addition to these tumour samples, 10 samples of normal oesophageal squamous epithelium adjacent to tumour were collected from Anyang Tumour Hospital for comparative study of CD117 expression. All patients provided written informed consent.

Twenty-two patients received adjuvant chemotherapy and eight patients received adjuvant radiotherapy after surgery. There were 36 patients who had recurrence or metastasis during the follow-up. The patients' clinical information and tumour parameters are listed in Table 1. All of the patients were followed until May 2004, except for 16 patients for whom the follow-up data were missing. Patient follow-up information was available for a minimum period of 10 years. A total of 83 (52.9%) patients died during the follow-up period; the median follow-up time for these patients was 61 months (range, 1–139 months). The median follow-up time for the 58 patients still alive was 124 months (range, 118–155 months).

**Table 1:** Clinicopathological features of 157 oesophageal SCC patients

Characteristics	*n* (%)
Gender	
Male	95 (60.5)

Female	62 (39.5)

Age (years)	
≤57	79 (50.3)

>57	78 (49.7)

Histological grade	
Well differentiated	59 (37.6)

Moderately differentiated	47 (29.9)

Poorly differentiated	51 (32.5)

Tumour location	
Upper	17 (10.8)

Middle	109 (69.4)

Lower	31 (19.7)

Tumour size (mm)	
<30	34 (21.7)

30–60	109 (69.4)

>60	14 (8.9)

Clinical stage	
I	3 (1.9)

II	96 (61.1)

III	11 (7.0)

IV	37 (23.6)

Unknown	10 (6.4)

T stage	
T1	5 (3.2)

T2	54 (34.4)

T3	70 (44.6)

T4	18 (11.4)

Tx	10 (6.4)

Lymph node metastasis	
Negative	90 (57.3)

Positive	57 (36.3)

Unknown	10 (6.4)

Distant metastasis	
No	120 (76.4)

Yes	37 (23.6)

CD117 expression	
Negative	110 (70.1)

Weakly positive	15 (9.6)

Moderately positive	15 (9.6)

Strongly positive	17 (10.8)

### Tissue microarray construction

Multi-tissue array blocks were made with the MTA-1 manual tissue arrayer (Beecher Instruments, Sun Prairie, WI, USA). Briefly, 4-μm sections from the routinely prepared primary tumour paraffin blocks were stained with H&E, and re-evaluated to confirm the diagnosis and to identify three representative tumour areas. Then, the related paraffin blocks were oriented and marked. From these blocks, tissue cores with a diameter of 0.6 mm were punched and arrayed in triplicate on a recipient paraffin block. After construction of the block was complete it was placed into a 40°C oven overnight to tighten the cylinders by slightly melting the paraffin. Four-micrometre sections of these tissue array blocks were cut, placed on Super-Frost Plus glass slides, and dried at 60°C in an oven for 2–4 h. These sections were used for immunohistochemical analysis. For those samples for which tissue array materials were not representative or not available, or only one core section was positive and the other two were negative on staining, paraffin-embedded conventional sections were used for additional immunohistochemical analyses.

### Immunohistochemistry

The Envision Plus detection system (Dako, Carpinteria, CA, USA) was used for detection of immunostaining. The sections were deparaffinized in xylene, and then microwaved in 10 mm citrate buffer (pH 6.0) to unmask the epitopes. Endogenous peroxidase activity was blocked by incubation with 0.03% hydrogen peroxide in methanol for 5 min. Sections were incubated with a polyclonal rabbit antihuman CD117 antibody (Dako, Glostrup, Denmark; code A4502, lot number 10037853; diluted 1:400) for 30 min at room temperature. After being gently rinsed three times with washing buffer, the sections were incubated with peroxidase-labelled polymer conjugated to goat antirabbit IgG (Dako) for 30 min before being stained for 5 min with 3,'3-diaminobenzidine tetrahydrochloride (DAB), counterstained with haematoxylin, dehydrated, and mounted in Diatex. A known CD117-positive seminoma was used as positive control, and the same concentration of non-immune rabbit IgG was applied as a negative control. Both controls gave satisfactory results.

### Scoring of immunohistochemical staining

Tumour cell immunoreactivity was scored according to both intensity and extent of staining. The extent of positivity was scored as follows: 0, no positive cells; 1, <10% positive cells; 2, 10–50% positive cells; and 3, >50% positive cells. The intensity was scored as follows: 0, no positive cells; 1, weak staining; 2, moderate staining; and 3, strong staining. Multiplying extent by intensity gave the immunohistochemical staining score (0, 1, 2, 3, 4, 6 or 9). For statistical analyses, a score of 0 was designated grade 0 (negative), a score of either 1 or 2 as grade 1 (weakly positive), a score of either 3 or 4 as grade 2 (moderately positive), and a score of either 6 or 9 as grade 3 (strongly positive).^17^ The evaluation was performed independently by two experienced investigators who were unaware of the related clinical information before conducting statistical analyses.

### Statistical analyses

Statistical analyses were performed using SPSS 17.0 (SPSS, Chicago, IL, USA). Pearson's *χ*^2^-test was used to analyse the correlation between clinicopathological features and CD117 expression. Overall survival was calculated from the date of diagnosis to the date of death or 1 May 2004. Survival curves were plotted according to the Kaplan–Meier method, and the log-rank test was used to determine significant differences among groups. Significant variables were further analysed using Cox regression (proportional hazard model) in the multivariate analyses. Spearman's correlation coefficient was used to evaluate the correlations between variable factors. A *P*-value of <0.05 was considered to be statistically significant.

## Results

### Immunostaining of CD117 in tumour samples

**Figure 1:**
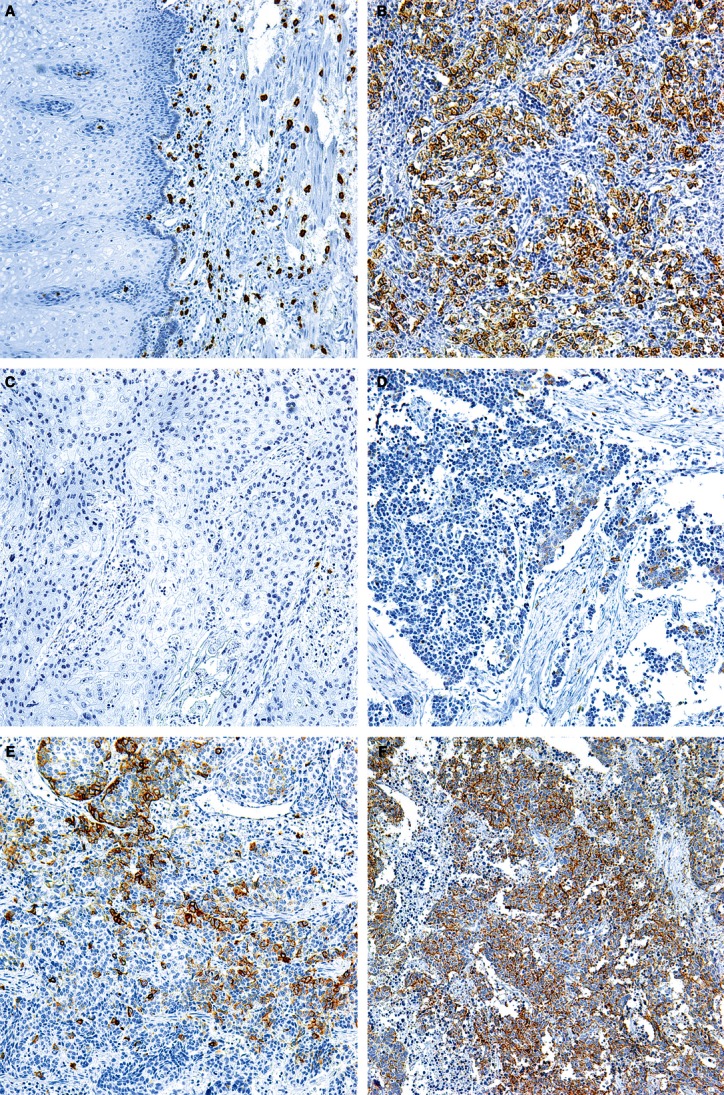
Immunohistochemistry for CD117 in normal oesophageal epithelia and oesophageal squamous cell carcinoma (SCC). **A**, Negative expression in normal oesophageal epithelium. **B**, Seminoma positive control. **C**, Negative expression in an oesophageal SCC. **D**, Weak positivity in an oesophageal SCC. **E**, Moderate positivity in an oesophageal SCC. **F**, Strong positivity in an oesophageal SCC.

Immunohistochemistry demonstrated no expression of CD117 in the 10 normal oesophageal epithelia adjacent to tumour (Figure 1A). In contrast, CD117 expression was observed in the cytoplasm and membrane of cancer cells. In total, 47 of the 157 tumours (29.9%) were positive for CD117 expression, and 110 of the tumours (70.1%) were negative (Figure 1C). Fifteen tumours (9.5%) were scored as grade 1 (weakly positive; Figure 1D), 15 (9.5%) as grade 2 (moderately positive; Figure 1E), and 17 (10.8%) as grade 3 (strongly positive; Figure 1F).

### Correlation between CD117 expression and clinicopathological features

The CD117 immunohistochemical findings were analysed for potential correlations with clinicopathological features in the 157 cases of oesophageal SCC. As summarized in Table 2, there were no significant correlations between CD117 expression and gender (*P* = 0.361), age (*P* = 0.356), histological grade (*P* = 0.835), tumour location (*P* = 0.192), or tumour size (*P* = 0.752). However, CD117 expression was significantly associated with T stage (*P* < 0.001), lymph node metastasis (*P* = 0.019), distant metastasis (*P* = 0.015), and clinical stage (*P* = 0.021). The correlation between CD117 expression and clinicopathological factors was further validated using Spearman correlation analysis. High CD117 expression correlated positively with higher T stage, lymph node metastasis, distant metastasis, and advanced clinical stage, with Spearman's correlation coefficients of 0.435, 0.194, 0.194, and 0.190, respectively.

**Table 2:** Correlations between tumour CD117 expression and clinical variables

		CD117 expression, *n* (%)			
				
Features	Total	Negative	Positive	*P*-value	χ^2^
Gender					
Male	95	64 (67.4)	31 (32.6)	0.361	0.833
		
Female	62	46 (74.2)	16 (25.8)		

Age (years)					
≤57	79	58 (73.4)	21 (26.6)	0.356	0.853
		
>57	78	52 (66.7)	26 (33.3)		

Histological grade					
Well differentiated	59	43 (72.9)	16 (27.1)	0.835	0.361
		
Moderately differentiated	47	32 (68.1)	15 (31.9)		
		
Poorly differentiated	51	35 (68.6)	16 (31.4)		

Tumour location					
Upper	17	11 (64.7)	6 (35.3)	0.192	3.299
		
Middle	109	81 (74.3)	28 (25.7)		
		
Lower	31	18 (58.1)	13 (41.9)		

Tumour size (mm)					
<30	34	24 (70.6)	10 (29.4)	0.752	0.570
		
30–60	109	75 (68.8)	34 (31.2)		
		
>60	14	11 (78.6)	3 (21.4)		

Clinical stage					
I/II	99	76 (76.8)	23 (23.2)	0.021	5.308
		
III/IV	48	28 (58.3)	20 (41.7)		

T stage					
T1/T2	59	56 (94.9)	3 (5.1)	<0.001	27.814
		
T3/T4	88	48 (54.5)	40 (45.5)		

Lymph node metastasis					
Negative	90	70 (77.8)	20 (22.2)	0.019	5.542
		
Positive	57	34 (59.6)	23 (40.4)		

Distant metastasis					
No	120	90 (75.0)	30 (25.0)	0.015	5.916
		
Yes	37	20 (54.1)	17 (45.9)		

Among the 36 patients who had recurrence or metastasis during follow-up, 16 patients were CD117-positive and the others were CD117-negative. The recurrence or metastasis rate in patients with CD117-positive tumours was 34.8%, whereas the rate in patients with CD117-negative tumours was 18.9% (*P* = 0.034). All of the 36 patients received chemotherapy after recurrence or metastasis. According to the Response Criteria in Solid Tumours (RECIST) guidelines, version 1.0,^18^ 18 patients, including five CD117-positive patients and 13 CD117-negative patients, were confirmed as having complete response (CR) or partial response (PR). Eighteen patients, including 11 CD117-positive patients and seven CD117-negative patients, had stable disease (SD) or progressive disease (PD). SD and PD were defined as resistance to chemotherapy. Chemotherapy resistance rates in the CD117-positive group and in the CD117-negative group were 68.8% and 35.0%, respectively (*P* = 0.044).

### Association of CD117 expression with progression-free survival and overall survival

**Figure 2:**
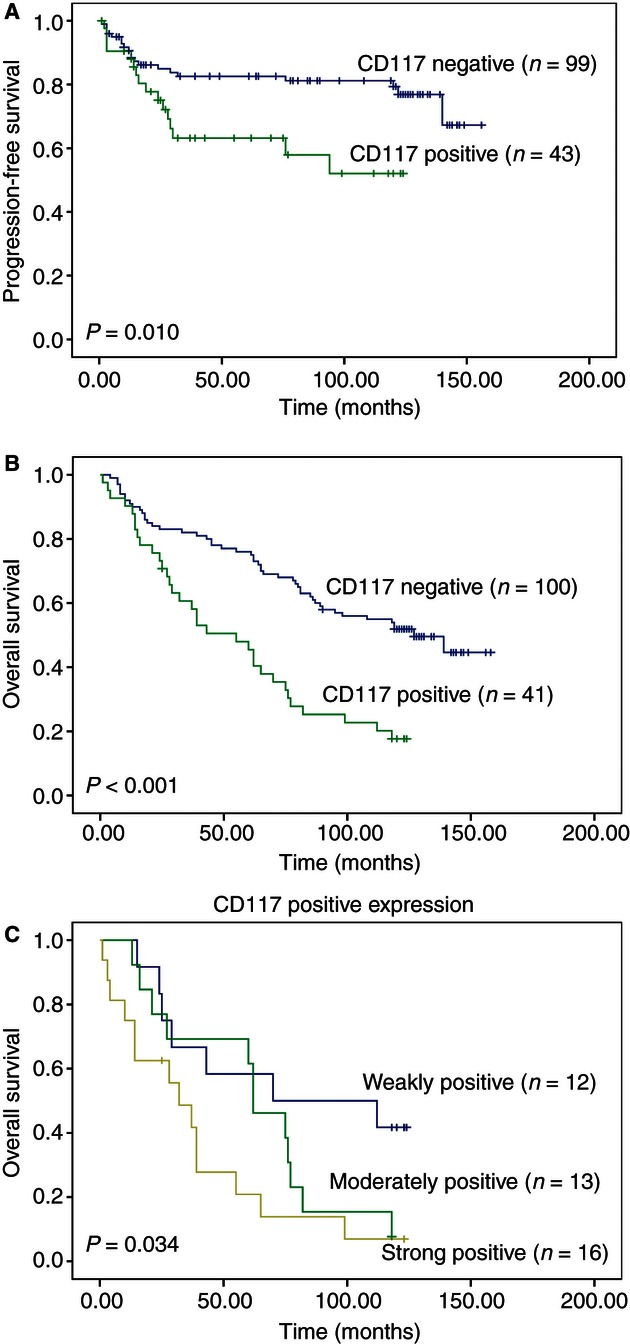
Univariate survival analyses according to cumulative Kaplan–Meier methods. Both progression-free survival (**A**) and overall survival (**B**) of patients with tumours expressing CD117 were shorter than those of patients with CD117-negative tumours. Median overall survival times of the patients with tumours showing weak, moderate and strong expression of CD117 were gradually shortened (**C**). *P*-values were determined by the log-rank test.

Progression-free survival was defined as the time from surgery until objective assessment of disease progression according to the RECIST guidelines or death. An analysis showed that mean progression-free survival times in the CD117-positive group and in the CD117-negative group were 80.3 months (95% CI 63.8–96.8) and 126.9 months (95% CI 115.4–138.4), respectively (*P* = 0.010) (Figure 2A).

In univariate analysis, CD117 expression was the most significant factor for the overall survival of oesophageal SCC patients (*P* < 0.001), followed by lymph node metastasis (*P* = 0.001), T stage (*P* = 0.002), clinical stage (*P* = 0.006), distant metastasis (*P* = 0.020), and histological grade (*P* = 0.027). There was a negative correlation between CD117 expression and overall survival, with a Spearman's correlation coefficient of −0.366 (*P* < 0.001). The median survival time of patients in the CD117-negative group was significantly longer than that of patients in the CD117-positive group (*P* < 0.001; Figure 2B). The median overall survival time was 127 months (95% CI 89.5–164.4) in the CD117-negative group, whereas it was only 55 months (95% CI 33.1–76.9) in the CD117-positive group. Furthermore, when patients with CD117-positive tumours were divided into three groups according to different staining scores, median overall survival times for those with tumours showing weak, moderate and strong expression of CD117 shortened progressively (*P* = 0.034; Figure 2C). There were no significant overall survival correlations with other clincopathological parameters, including gender, age, tumour location, and tumour size (log-rank test, *P* ≥ 0.05).

Multivariate analysis was performed to determine independent prognostic factors for overall survival in oesophageal SCC patients. Our results indicated that CD117 expression (*P* = 0.002), distant metastasis (*P* = 0.023) and lymph node metastasis (*P* = 0.045) had independent prognostic impacts in the Cox model (Table 3), whereas histological grade (*P* = 0.251) and T stage (*P* = 0.301) did not.

**Table 3:** Multivariate analysis of overall survival in 157 patients with oesophageal squamous cell carcinoma

Factors	HR	95% CI	*P*
CD117 expression*	2.293	1.370–3.836	0.002

Distant metastasis†	1.353	1.043–1.756	0.023

Lymph node metastasis‡	1.912	1.016–3.599	0.045

Histological grade§	0.662	0.328–1.338	0.251

T stage¶	1.348	0.765–2.375	0.301

HR, hazard ratio; 95% CI, 95% confidence interval.

* Positive CD117 expression versus negative CD117 expression.

† With distant metastasis versus without distant metastasis.

‡ Positive lymph nodes versus negative lymph nodes.

§ Poorly differentiated versus moderately differentiated versus well differentiated.

¶ T3/T4 versus T1/T2.

**Figure 3:**
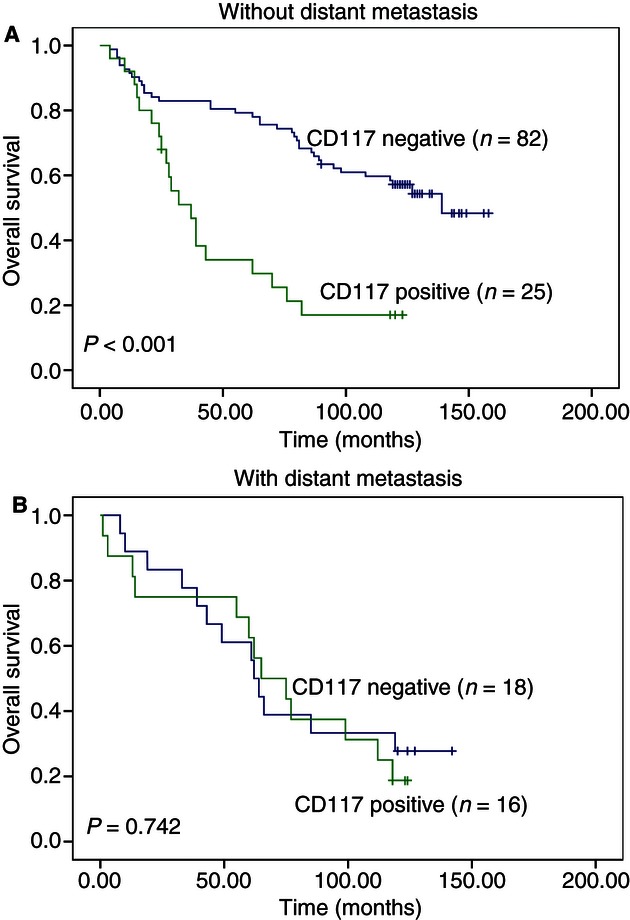
Overall survival analyses in different patient subgroups: Kaplan–Meier survival analysis of overall survival in patients without distant metastases (**A**) and with distant metastases (**B**) according to tumour CD117 expression. *P*-values were determined by the log-rank test.

In addition, we investigated the prognostic value of CD117 expression in different subgroups according to distant metastasis. CD117 overexpression was shown to further stratify survival in patients without distant metastases (Figure 3A). Among these patients, those who had CD117-expressing tumours had shorter survival than those with CD117-negative tumours (*P* < 0.001); the median survival time for the former was 50.0 months (95% CI 34.7–65.4), and for the latter was 111.4 months (95% CI 99.1–123.7). Conversely, in the subgroup of patients with distant metastases, overall survival for those with CD117-positive and those with CD117-negative tumours showed no significant difference (*P* = 0.742; Figure 3B).

## Discussion

In this study, we have shown, for the first time, the expression patterns and prognostic value of CD117 in oesophageal SCC. We found that CD117 expression was linked strongly to adverse prognostic factors, including clinical stage, T stage, lymph node metastasis, and distant metastasis, as well as progression-free survival and overall survival, as confirmed by multivariate analysis. Furthermore, we observed that CD117 expression was strongly associated with poorer survival in patients without distant metastases.

We detected CD117 expression in 29.9% of oesophageal SCC tumours, which was lower than the CD117-positive rate of 37–87.7% in small-cell lung cancer, but within the CD117-positive rate of 26–73% in ovarian carcinoma.^19^–^24^ We found that there was no CD117 expression in the 10 normal oesophageal epithelia adjacent to tumours. This finding showed that CD117 expression was up-regulated in oesophageal SCC tissues, suggesting that aberrant expression of CD117 may be involved in the pathogenesis of oesophageal SCC.

Several authors have reported that CD117 (c-kit) bound by SCF could enhance autocrine growth stimulation in a variety of solid tumours, including small-cell lung cancer, breast cancer, and intrahepatic cholangiocarcinoma.^25^–^27^ Moreover, the stem cell factor–CD117 pathway plays a vital role in promoting angiogenesis, which is necessary for tumour growth.^28^ It was also confirmed previously that, through binding to stem cell factor, CD117 plays a critical role in stimulating invasion and metastasis in several types of cancer, including breast, prostate, pancreatic and salivary adenoid cystic cancers.^29^–^32^ CD117 activation triggers multiple signal transduction pathways, such as the PI3K–Akt, Ras–MAP kinase and JAK–STAT pathways.^33^ Yasuda *et al*.^34^ demonstrated that stem cell factor–CD117 signalling enhanced proliferation and invasion in CD117-positive colorectal cancer cell lines, mainly through the PI3K–Akt pathway. In line with these experimental results, we found that CD117 expression was positively correlated with higher T stage, lymph node metastasis, and distant metastasis. This finding may suggest that CD117 up-regulation is an important factor contributing to invasion and metastasis in patients with oesophageal SCC.

In this study, patients with CD117-positive tumours had shorter progression-free survival and overall survival than those with CD117-negative tumours. This result was confirmed by multivariate analysis using the Cox model to identify prognostic factors. In keeping with our results, El-Serafi *et al*.^35^ found that CD117 over-expression was significantly correlated with reduced disease-free survival in stage II colorectal cancer patients. Micke *et al*.^19^ examined the influence of CD117 on survival in extended-stage small-cell lung cancer, and found that patients with CD117 expression and a minor response to chemotherapy had shorter survival times than those with CD117-negative tumours.

Combining systemic chemotherapy and local-regional surgery can improve survival in patients with operable oesophageal cancer.^36^ However, some researchers have reported that CD117 overexpression and/or activation significantly correlated with resistance to chemotherapy in patients with ovarian carcinoma and malignant mesothelioma.^37^,^38^ Moreover, Chau *et al*.^39^ confirmed that CD117 mediated resistance to chemotherapy through activation of Wnt–β-catenin–ATP-binding cassette G2 signalling. We observed that the chemoresistance rate in the CD117-positive group was higher than that in the CD117-negative group. On the basis of these results, we propose that resistance to chemotherapy may be a reason for shorter survival in CD117-positive oesophageal SCC patients.

In recent years, substantial experimental evidence has been generated in support of the role of a small population of self-renewing cells that may sustain malignant growth.^40^ This subpopulation has been termed tumour-initiating cells or cancer stem cells (CSCs). In contrast to other tumour cells, CSCs are drug-resistant, and show the ability to self-renew and differentiate.^41^,^42^ It has been shown that CD117 was up-regulated in lung CSCs and ovarian CSCs, and mediated chemoresistance.^39^,^43^ It is likely that CD117 up-regulates the stemness of tumour cells and is involved in chemoresistance in oesophageal SCC as well, which may provide an explanation for our observation that the nearly 30% of oesophageal SCC patients whose tumours expressed CD117 had shorter survival times than those with CD117-negative tumours.

CD117 expression in the subgroup of patients without distant metastases showed the same clinical outcome as that of the whole group; that is, it was negatively associated with overall survival (*P* < 0.001). However, CD117 expression in the subgroup of patients with distant metastases showed no effect on overall survival. As discussed above, CD117 may play an important role in tumour invasion and metastasis. It is possible that the CD117 expression in the primary tumours endowed them with higher capability for invasion and metastasis, and therefore led to shorter survival times. However, it is difficult to explain why CD117 positivity in the group with distant metastases was not associated with overall survival, as shown in the non-distant metastasis group. There are at least two possible explanations for these contradictory findings. First, there might be more powerful secondary genetic alterations other than CD117 alone in the metastatic tumours, which may mask the role of CD117, so that the CD117 survival effect was less apparent. Another possibility may be the small number of CD117-positive tumours included in our study, which may limit our findings.

In summary, CD117 expression can be detected in approximately one-third of oesophageal SCCs, and its expression predicts poorer survival. CD117 expression may therefore be a useful prognostic marker for identifying an aggressive molecular subgroup of oesophageal SCC, and may serve as an attractive therapeutic target in oesophageal SCC patients. Our results suggest that functional studies are needed to further disclose the molecular mechanisms of CD117 expression involved in the progression of oesophageal SCC. It should be noted that our present study is based on a small number of cases, which may limit our conclusions. Therefore, retrospective and prospective studies of the clinical significance of CD117 expression in larger numbers of SCCs are warranted.
